# Chromosome 1 trisomy confers resistance to aureobasidin A in *Candida albicans*

**DOI:** 10.3389/fmicb.2023.1128160

**Published:** 2023-03-17

**Authors:** Lijun Zheng, Yi Xu, Yubo Dong, Xiaowen Ma, Chen Wang, Feng Yang, Liangsheng Guo

**Affiliations:** ^1^Department of Ultrasound Medicine, The Second Affiliated Hospital of Soochow University, Suzhou, China; ^2^Department of Pharmacy, The 960 Hospital of PLA, Jinan, China; ^3^Department of Pharmacology, Shanghai Tenth People’s Hospital, Tongji University School of Medicine, Shanghai, China; ^4^Department of Obstetrics and Gynecology, The Second Affiliated Hospital of Soochow University, Suzhou, China

**Keywords:** *Candida albicans*, aureobasidin A, sphingolipid biosynthesis, aneuploidy, antifungal drugs

## Abstract

**Introduction:**

*Candida albicans* is a prevalent opportunistic human fungal pathogen. However, there are currently very few antifungal treatments available. Inositol phosphoryl ceramide synthase is an essential and fungal-specific protein that also provides a novel and promising antifungal target. Aureobasidin A is a widely used inhibitor of inositol phosphoryl ceramide synthase, however the mechanism of resistance to aureobasidin A is largely unknown in pathogenic fungi.

**Methods:**

Here we investigated how *C. albicans* adapted to low and high concentrations of aureobasidin A.

**Results and discussions:**

We identified trisomy of chromosome 1 as the predominant mechanism of rapid adaptation. Resistance to aureobasidin A was unstable because of the inherent instability of aneuploids. Importantly, chromosome 1 trisomy simultaneously regulated genes which were associated with aureobasidin A resistance that are on this aneuploid chromosome as well as on other chromosomes. Furthermore, the pleiotropic effect of aneuploidy caused altered resistance not only to aureobasidin A but also to other antifungal drugs including caspofungin and 5-flucytosine. We posit aneuploidy provides a rapid and reversible mechanism of development of drug resistance and cross resistance in *C. albicans*.

## Introduction

In recent years, the increase in advanced transplant procedures, the use of immunosuppressive therapies and the pandemic spread of HIV has resulted in an increase in the population of immunocompromised patients (Benedict et al., 2019), and the frequency of opportunistic fungal infections, especially invasive mycoses, has also increased significantly (Pfaller and Diekema, 2004). The most prevalent fungal infections are aspergillosis and candidemia. *Candida* species are the fourth most common cause of nosocomial bloodstream infections in the United States (Pfaller and Diekema, 2007, 2010). *Candida albicans* is the predominant cause of candidiasis (Pappas et al., 2018).

Fungi are eukaryotes and share many similar metabolic pathways and essential cellular machinery with humans. These inherent similarities have severely limited the antifungal drugs to only four classes: echinocandins, azoles, polyenes and flucytosine (Fisher et al., 2022). Alarmingly, multi-drug resistance in *C. glabrata* and *C. auris* has been reported (Arendrup and Patterson, 2017). In this context, identifying novel antifungal targets and the development of new antifungal agents are urgently needed.

Sphingolipids are complex lipids that have a sphingoid base as a backbone, to which a fatty acid and variable head groups are attached. Sphingolipids are found on both the inner and outer membrane of eukaryotic cells. They are essential components of eukaryotic cell membranes and play a pivotal role in a variety of biological processes such as cell division, heat stress response, acid/alkaline tolerance, morphogenesis, signal transduction, endocytosis and apoptosis (Singh and Del Poeta, 2016; McEvoy et al., 2020). The major fungal sphingolipids are inositol phosphoryl-ceramides (IPCs) and glucosylceramide (GlcCer). IPCs are the product of inositol phosphoryl ceramide synthase (Ipc1). Ipc1 is an essential fungal-specific enzyme for fungal cell growth and has no mammalian homolog (Denny et al., 2006). Therefore, Ipc1 is an ideal potential antifungal target.

Aureobasidin A (AbA) is one of the most widely studied IPC inhibitors. AbA is a cyclic depsipeptide isolated from the fungus *Aureobasidium pullulans* R106 (Ikai et al., 1991). It is fungicidal. AbA strongly inhibits Ipc1 almost exclusively in yeasts, including some notorious pathogenic fungi such as *C. albicans, and Cryptococcus neoformans* (Tan and Tay, 2013; Teymuri et al., 2021). In yeasts, Ipc1 is encoded by the gene *AUR1*. The mechanisms of resistance to AbA have been best studied in the model yeast *Saccharomyces cerevisiae*. In most cases, resistance to AbA is caused by mutation or overexpressoin of *AUR1* (Heidler and Radding, 1995; Hashida-Okado et al., 1996). Overexpression of *PDR16* also confers resistance to AbA putatively via reducing the effectiveness of AbA against Ipc1 (Katsuki et al., 2018). In addition to *AUR1*, deletion of other genes encoding sphingolipid-metabolizing enzymes also causes AbA resistance (Oh et al., 1997; Schorling et al., 2001; Tani and Kuge, 2010). AbA is likely a substrate of the ATP-binding cassette (ABC) transporters in *S. cerevisiae*. Overexpression of *YOR1*, which encodes plasma membrane ABC transporter, confers resistance to AbA (Ogawa et al., 1998). However, reports of genetic mechanisms of resistance to AbA in pathogenic fungi are still lacking.

In this study, we investigated genetic mechanisms of adaptation to both sub-inhibitory and lethal amount of AbA in *C. albicans*. We found formation of Chromosome 1 trisomy (Chr1 × 3) was the predominant adaptation strategy. In the absence of stress, Chr1 × 3 mutants were unstable and spontaneously reverted to euploids with resistance to AbA concomitantly lost. We found *C. albicans PDR16* and *AUR1* genes were haploinsufficient. Heterozygous deletions of *PDR16* and *AUR1* caused hypersensitivity to AbA. Chr1 × 3 simultaneously upregulated expression of genes on Chr1 including *PDR16*, as well as genes on other chromosomes including *AUR1*. Furthermore, Chr1 × 3 conferred pleiotropic effects on susceptibility to other antifungals including decreased resistance to caspofungin and increased resistance to 5-flucytosine. Therefore, we posit Chr1 × 3 provides a rapid mechanism of reversible adaptation to AbA via simultaneously upregulating genes on and outside of the aneuploid chromosome.

## Materials and methods

### Strains and growth conditions

Strains used in this study are listed in Supplementary Table 1. Primers are listed in Supplementary Table 2. Drugs were dissolved in dimethyl sulfoxide (DMSO) and stored at –20°C.

*C. albicans* lab strain SC5314 was used as wild type in this study. Stock culture was preserved in 25% glycerol and maintained at –80°C. Cells were grown in Yeast extract-Peptone-Dextrose (YPD) media (1% [w/v] yeast extract, 2% [w/v] peptone and 2% [w/v] D-glucose) at 37°C in a shaking incubator at 150–200 rpm. For solid medium, 2% [w/v] agar was added. For the selection of gene knockout strains, YPD agar containing 400 μg/ml nourseothricin (Werner BioAgents) medium was used (YPD + NAT).

### Growth curves

Approximately 2.5 × 10^3^ cells/ml of SC5314 in 150 μl YPD with or without AbA were incubated in a 96 well plate at 37°C. OD_595_ was monitored in a Tecan plate reader (Infinite F200 PRO, Tecan, Switzerland) at 15 min time intervals for 24 h. Data are represented as the mean ± SD of three biological replicates.

### Spot assay

Cells were suspended in distilled water and adjusted to 1 × 10^7^ cells/ml. 3 μl of 10-fold serial dilutions were spotted on YPD plates with or without drugs (control) at 37°C and photographed after 2 days.

### Obtaining mutants using lethal amount of aureobasidin A

Cells were suspended in distilled water and adjusted to 1 × 10^7^ cells/ml. 100 μl of cell suspension were on YPD plates supplemented with 20 ng/ml AbA. The plates were incubated at 37°C for 3 days. 18 mutants were randomly chosen. The mutants were streaked onto YPD plates and incubated at 37°C for 36 h. For each mutant, 4–6 colonies of similar size were selected and frozen in 1 ml of 25% glycerol at –80°C.

### Short time exposure to sub-inhibitory aureobasidin A

Approximately 1 × 10^3^ cells/ml of SC5314 was grown in YPD broth (control) or YPD broth supplemented with 5 ng/ml AbA (test). After 24 h of shaking at 37°C, 10 μL of the culture was transferred to 1 ml YPD broth containing the same AbA concentration. After 24 h, the culture was centrifuged at 3,000 rpm for 1 min, and resuspended in distilled water. Then it was diluted with distilled water. Approximately 200 cells were spread onto YPD plates. The plates were incubated at 37°C for 24 h. Randomly 40 colonies from each plate were tested for resistance to AbA.

### Gene deletions

Gene deletions were constructed using the *NAT1* flipper cassette as described previously (Yang et al., 2019). Briefly, approximately 500 bp of upstream and downstream region of the target gene was amplified using SC5314 genomic DNA as template. The 3’ end of the upstream forward primers overlapped with the 5’ end of the *NAT1* flipper; the 5’ end of the downstream reverse primers overlapped with the 3’ end of the *NAT1* flipper. The amplicon and plasmid pJK863 were used as templates to fuse the upstream region to the 5’ region of the cassette, and the downstream region to the 3’ region of the cassette. The fusion products were transformed into *C. albicans* following the lithium acetate method (Wilson et al., 2000), and transformants were selected on YPD plates supplemented with 400 μg/ml nourseothricin (NAT). Positive transformants were confirmed by diagnostic PCRs with primers that annealed outside the flanking homology regions. All primer sequences are listed in Supplementary Table 2.

### DNA-seq

Test strains were grown on YPD-agar plates. Randomly 25–30 colonies with similar sizes were collected. Total DNA extraction and genomic DNA library preparation were performed as described previously (Yang et al., 2021d). The final libraries were sequenced by BGISEQ-500. Raw fastq files were uploaded to YMAP (version 1.0)^1^ (Abbey et al., 2014). Read depth was plotted as a function of chromosome position using the Assembly 22 version of the SC5314 reference genome.^2^

### RNA-seq

RNA-seq was performed as described previously (Yang et al., 2021b). Strains were streaked onto YPD plates from the –80°C freezer. After 36 h incubation at 37°C, several colonies of similar sizes were chosen. Colonies were suspended in distilled water and adjusted to 1 × 10^4^ cells/ml. 100 μl of cell suspension were spread on YPD plates. The plates were incubated at 37°C for 36 h. Cells were collected by centrifugation, washed and flash frozen in liquid nitrogen. Total RNA extraction and purification, library construction, and sequencing were performed as described in Yang et al. (2013).

## Results

### Measuring susceptibility of *Candida albicans* lab strain SC5314 to aureobasidin A

**FIGURE 1:**
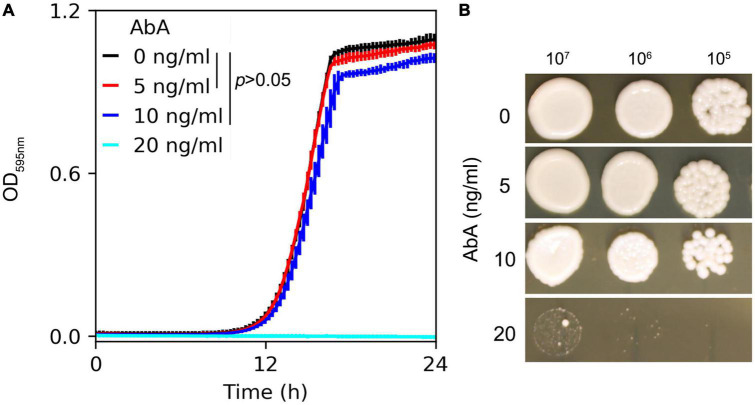
Susceptibility of *C. albicans* to aureobasidin A. *C. albicans* lab strain SC5314 was grown in YPD broth **(A)** or on YPD plates **(B)** supplemented with aureobasidin A. In panel **(A)** optical density at 595 nm (OD_595_) was measured every 15 min for 24 h at 37°C using a Tecan plate reader (Infinite F200 PRO, Tecan, Switzerland). Data are represented as the mean ± SD of three biological repeats. In panel **(B)**, 3 μl of 10-fold serial dilutions were spotted on YPD plates. Drug concentrations are shown in the figure. The plates were incubated at 37°C for 48 h then photographed.

Susceptibility of the *C. albicans* lab strain SC5314 to AbA was measured in YPD broth and on YPD agar supplemented with AbA. In the liquid medium, growth in the presence of 5 ng/ml or 10 ng/ml of AbA did not significantly inhibit growth compared to the in YPD broth without AbA [*p* > 0.05, one-way ANOVA (ANalysis Of VAriance) with *post hoc* Tukey HSD (Honestly Significant Difference)]. 20 ng/ml of AbA completely inhibited growth (Figure 1A). On the plates, growth was also inhibited by 20 ng/ml of AbA (Figure 1B). Therefore, we chose 5 ng/ml and 20 ng/ml of AbA as sub-inhibitory and inhibitory concentrations, respectively, for further studies.

### Lethal amount of aureobasidin A selects for chromosome 1 trisomy mutants in *Candida albicans*

**FIGURE 2:**
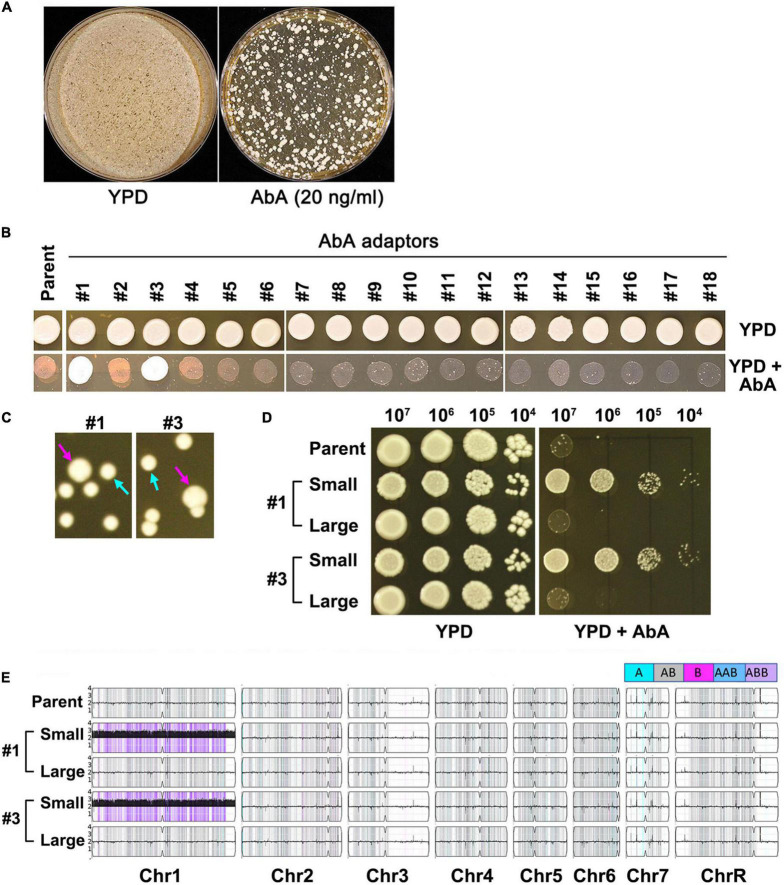
Lethal amount of aureobasidin A selects unstable aneuploid mutants. Approximately 1 million cells of SC5314 were spread on YPD plate supplemented with 20 ng/ml aureobasidin A or without drug (control). The plates were incubated at 37°C for 3 days **(A)**. Randomly 18 mutants were chosen. Spot assay was performed to compare level of resistance between mutants and the parent **(B)**. Two resistant mutants (#1 and #3) were spread on YPD plates. Cyan arrows indicate small colonies and magenta arrows indicate large colonies **(C)**. Both small and large colonies were tested for resistance to aureobasidin A **(D)** and were sequenced **(E)**. The karyotypes were generated using Ymap (Abbey et al., 2014). Allele frequencies are color coded: homolog “a” is cyan, homolog “b” is magenta and heterozygous alleles are gray.

Approximately 1 million cells of SC5314 were spread on YPD plate supplemented with 20 ng/ml AbA. After 3 days of incubation at 37°C, randomly 18 colonies (mutants) were chosen (Figure 2A). Spot assay indicated only 2 (#1 and #3) of the 18 mutants (#1-#18) grew better than the parent in the presence of AbA (Figure 2B). However, when grown on YPD plates in the absence of AbA, these 2 mutants were unstable, exhibiting mostly small sized colonies (indicated by cyan arrows) and a few large colonies (indicated by magenta arrows) (Figure 2C). When tested for resistance to AbA, only the small colonies were resistant. The large colonies were not (Figure 2D). Whole genome sequencing indicated in both mutants the small colonies were Chr1 × 3 and both had the B homolog duplicated (ABB), and the large colonies were euploid (Figure 2E). Therefore, exposure to lethal amount of AbA selected for resistant mutants which were Chr1 × 3. The resistance was unstable. Loss of aneuploidy caused concomitant loss of AbA resistance.

### Short-term exposure to sub-inhibitory aureobasidin A selects for chromosome 1 trisomy mutants in *Candida albicans*

**FIGURE 3:**
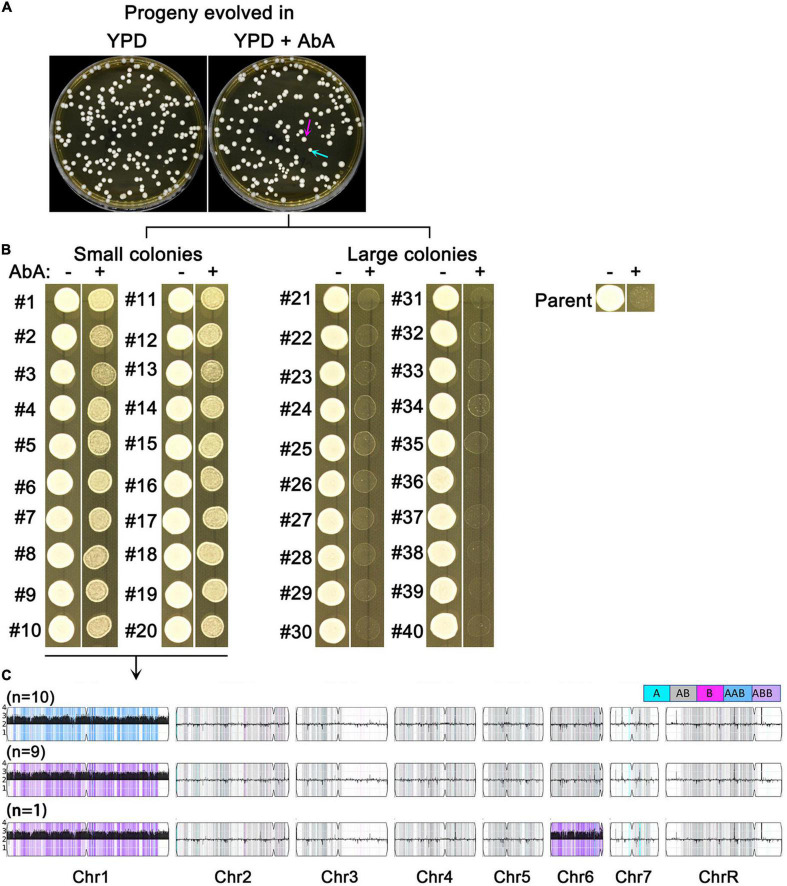
Sub-inhibitory amount of aureobasidin A selects aneuploid mutants. SC5314 was grown in YPD broth supplemented with or without 5 ng/ml aureobasidin A. After 48 h, the cultures were washed and diluted using distilled water. Approximately 200 cells were spread on YPD. The plates were photographed after 24 h incubation at 37°C. Cyan arrow indicates small colonies and magenta arrow indicates large colonies **(A)**. Randomly 20 small and 20 large colonies from the drug evolved culture were tested for resistance to aureobasidin A **(B)**, and all the 20 resistant mutants were sequenced **(C)**.

We asked if short term growth in sub-inhibitory AbA was sufficient to select for resistant mutants. SC5314 was grown in YPD broth or YPD broth supplemented with 5 ng/ml AbA. After 48 h, the cultures were washed, diluted and plated onto YPD. Surprisingly, the colonies (mutants) from the AbA-evolved culture showed obvious size variations: 117 mutants were large (indicated by magenta arrows) and 38 mutants were small (indicated by cyan arrows). In contrast, mutants from the YPD-evolved culture did not show colony size variations and the size was similar to the large mutants from the AbA-evolved culture (Figure 3A).

Randomly, 40 mutants of the YPD-evolved progeny were tested by spot assay for resistance to AbA. None of them gained resistance (data not shown). Randomly 20 small mutants and 20 large mutants from the AbA-evolved progeny were tested. All the 20 small mutants (#1-#20) were more resistant than the parent to AbA. None of the large mutants (#21 – #40) were resistant (Figure 3B).

All the 20 resistant mutants were sequenced, and all had Chr1 × 3: 19 had Chr1 × 3 only, and 1 mutant had Chr1 × 3 + Chr6 × 3. Among them, 10 had AAB and 10 had ABB of Chr1. Therefore, there was no homolog bias in the Chr1 × 3 mutants (Figure 3C).

Thus, 48 h exposure to sub-inhibitory AbA was sufficient to select for resistant mutants. The resistance was due to amplification of Chr1. There was no biased homolog duplication in the Chr1 × 3 mutants.

### Chr1 trisomy conferred resistance to aureobasidin A via up-regulating *PDR16* and *AUR1*

**FIGURE 4:**
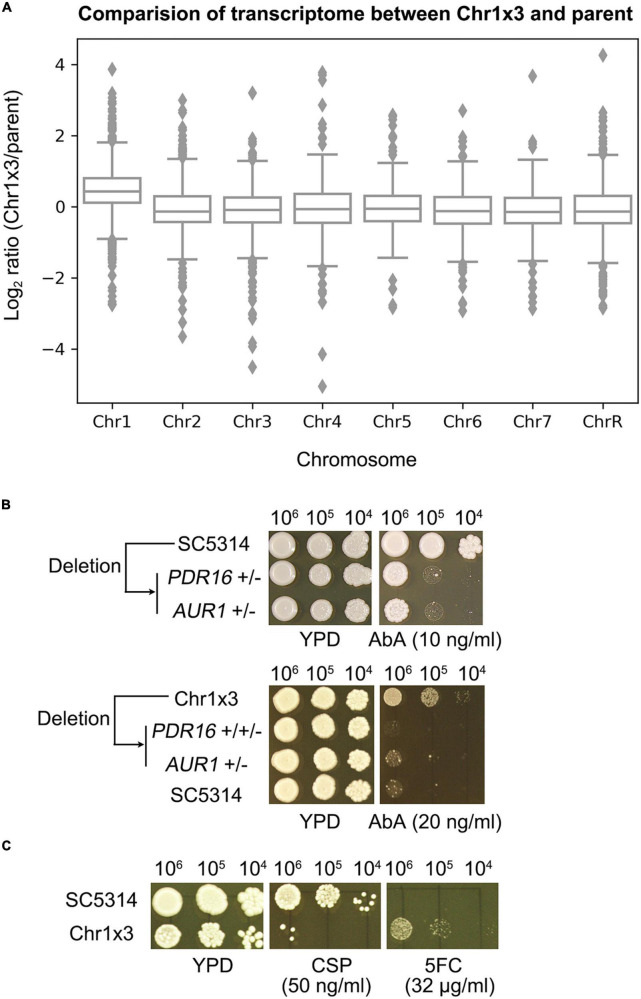
Chromosome 1 trisomy has pleiotropic effect on whole genome transcription and antifungal resistance profile. Transcriptome of one Chr1 trisomy mutant was compared to the diploid wild type strain. Cells were grown on YPD plates at 37°C. Log_2_ ratio of genes expression levels were plotted as a function of chromosome position **(A)**. Strains with deletions of one allele of *PDR16* or *AUR1* were compared to wild type diploid strain parent SC5314, or a Chr1 × 3 parent, for resistance to aureobasidin A (Ab) **(B)**. Pleiotropic effect of Chr1 trisomy on antifungal resistance was tested by spot assay on YPD plates supplemented with caspofungin (CSP) or 5-flucytosine (5FC) **(C)**. In panels **(B,C)**, the plates were incubated at 37°C for 48 h then photographed.

In the *C. albicans* genome, *PDR16* is on Chr1 and *AUR1* resides on Chr5. We asked why Chr1 × 3 caused resistance to AbA. The transcriptome of one Chr1 × 3 mutant was compared to the wild type strain. The expression of genes on Chr1 were generally up-regulated in the Chr1 × 3 mutant, including *PDR16* (Figure 4A and Supplementary Table 3). Chr1 × 3 also up-regulated genes on other chromosomes, including *AUR1* (Figure 4A and Supplementary Table 3). We found *PDR16* and *AUR1* were haploinsufficient. Deletion of one allele of *PDR16* or *AUR1* in SC5314 caused hypersensitivity to AbA. Deletion of one allele of *PDR16* or *AUR1* in a Chr1 × 3 mutant also caused loss of AbA resistance (Figure 4B). Therefore, Chr1 × 3 caused resistance to AbA via simultaneously up-regulating genes on the aneuploid chromosome (such as *PDR16*) as well as genes on euploid chromosomes (such as *AUR1*) which were associated with AbA resistance.

Furthermore, we asked if Chr1 × 3 had pleiotropic effects on the aneuploid cells. Susceptibility of Chr1 × 3 mutant was compared to parent by spot assay. Chr1 × 3 caused hypersensitivity to caspofungin (CSP). *GSC1* and *GSL1*, which encode the target protein β-1,3-glucan synthase of CSP, are on Chr1. RNA-seq indicated expression of *GSC1* was compensated to diploid level. *GSL1* was significantly up-regulated in Chr1 × 3, however, expression of *GSL2*, which also encodes a subunit of the β-1,3-glucan synthase, was significantly down-regulated (Supplementary Table 3). Deletion of the PKC pathway kinase genes *MKK2* and *MKC1*, and deletion of *CMP1* and *CNB1*, which encode subunits of calcineurin, cause hypersensitivity to CSP (Yang et al., 2021b). Here we found expression of *MKK2* and *MKC1* were significantly down-regulated in Chr1 × 3, but expression of *CMP1* or *CNB1* was not significantly different (Supplementary Table 3). In addition to altered susceptibility to CSP, Chr1 × 3 also conferred resistance to 5-flucytosine (5FC) (Figure 4C). RNA-seq indicated *FCA1* gene was significantly down-regulated in Chr1 × 3, other genes associated with 5FC resistance, such as *FUR1*, *FCY2*, *FCY21*, were not differentially expressed (Supplementary Table 3).

## Discussion

Gene copy number variation, mainly via aneuploidy, is a prevalent mechanism of rapid adaptation to stresses in *C. albicans* (reviewed in Tsai and Nelliat, 2019). Although *C. albicans* can exist as a haploid (Hickman et al., 2013), most *C. albicans* strains are diploids with 8 pairs of chromosomes (Jones et al., 2004). *C. albicans* is tolerant to aneuploidy. Each chromosome can be trisomic, but specific aneuploidy is always selected for by particular stressors and usually causes resistance to that stress (Yang et al., 2021d). Furthermore, at least in yeasts, aneuploidy typically causes proportional alterations of gene transcription and in most cases translation, therefore, aneuploidy has the potential of causing cross-resistance to unrelated stresses (Yang et al., 2013, 2019, 2021a,b,c).

In this study, we found Chr1 × 3 formation was the predominant mechanism of rapid adaptation to both sub-inhibitory and lethal amount of AbA in *C. albicans*. Genetic mutations of *AUR1* and *PDR16*, which are known genes associated with resistance to AbA, were not detected in the mutants. The loss of the extra copy of Chr1 was accompanied by loss of AbA resistance. Therefore, copy number of Chr1 was the mechanism of altered AbA resistance. We also found *PDR16* and *AUR1* genes were dosage-sensitive genes. Deletion of one allele was sufficient to cause hypersensitivity to AbA. In the mutants, *PDR16* is on the aneuploid chromosome and *AUR1* is on euploid chromosome. Interestingly, Chr1 × 3 simultaneously up regulated both *PDR16* and *AUR1*. Therefore, Chr1 × 3 had pleiotropic effects on the whole genome transcription and phenotypes. As a result, we found Chr1 × 3 not only caused AbA resistance, it also conferred resistance to 5FC and hypersensitivity to CSP.

In the *C. albicans* genome, three genes encode subunits of the target protein β-1,3-glucan synthase of CSP: *GSC1*, *GSL1* and *GSL2*. *GSC1* and *GSL1* are on Chr1 and *GSL2* is on ChrR. Compared to the euploid parent, in Chr1 × 3 mutant, transcription of *GSL1* was up regulated and transcription of *GSL2* was down regulated. Imbalanced expression level of genes encoding subunits of protein complex always leads to misassembly of the complex (Cardarelli et al., 2011). This mechanism might explain why Chr1 × 3 mutant was hypersensitive to CSP.

5FC is a prodrug. Upon uptake into fungal cells via the cytosine permease (encoded by *FCY2* and *FCY21*), it is converted into toxic 5-fluorouracil (5FU) by cytosine deaminase (encoded by *FCA1*). 5FU is then further processed by uracil phosphoribosyltransferase (encoded by *FUR1*), and the product inhibits both DNA and protein synthesis. Loss of function mutations of genes involved in the uptake and intracellular metabolism of 5FC have been associated with resistance in clinical *Candida* (Hope et al., 2004; Papon et al., 2007) and *Cryptococcus* isolates (Whelan, 1987; Billmyre et al., 2020; Chang et al., 2021). Here we found *FCA1* was down regulated in Chr1 × 3 mutant. *FUR1*, *FCY2* and *FCY21* were not differentially expressed. Therefore, decreased expression of *FCA1* might be the cause of resistance to 5FC.

## Conclusions

In summary, this study indicates aneuploidy is the major mechanism of rapid adaptation to AbA in *C. albicans*. Aneuploidy directly regulates genes on the aneuploidy chromosome and indirectly regulates genes on the euploid chromosome. The pleiotropic effect of aneuploidy has the potential of causing cross resistance to antifungal drugs.

## Data availability statement

The datasets presented in this study can be found in online repositories. The names of the repository/repositories and accession number(s) can be found below: https://www.ebi.ac.uk/arrayexpress/, E-MTAB-8942; https://www.ebi.ac.uk/arrayexpress/, E-MTAB-12400; and https://www.ebi.ac.uk/arrayexpress/, E-MTAB-12403.

## Author contributions

FY and LG analyzed the data. FY wrote the manuscript. LZ and YX carried out the research. YD, XM, and CW helped to develop the experimental idea and design. YX and LG funded the experiments. All authors approved the submitted version.

## Footnotes

1
http://lovelace.cs.umn.edu/Ymap/

2
http://www.candidagenome.org/download/sequence/C_albicans_SC5314/Assembly22/current/C_albicans_SC5314_A22_current_chromosomes.fasta.gz
