# Supplementary material for: Chromosome 1 trisomy confers resistance to aureobasidin A in Candida albicans
Source: Front Microbiol. 2023 Mar 17;14:1128160. doi: 10.3389/fmicb.2023.1128160 (PMC10063858; doi:10.3389/fmicb.2023.1128160)
Supplement: Supplementary file 1 [file Table_1.DOCX]

Table S1. Strains used in this study.

| Strain | Genotype | Parent | Source |
| --- | --- | --- | --- |
| SC5314 | Wild type |  | (1) |
| YCA347 | PDR16/pdr16::NAT1 flp | SC5314 | This study |
| YCA350 | AUR1/aur1::NAT1 flp | SC5314 | This study |
| YCA1041 | PDR16/PDR16/pdr16::NAT1 flp | Chr1x3 | This study |
| YCA1042 | AUR1/aur1::NAT1 flp | Chr1x3 | This study |

**References**

1. Noble SM, Johnson AD. 2005. Strains and strategies for large-scale gene deletion studies of the diploid human fungal pathogen Candida albicans. Eukaryot Cell 4:298-309.
