# Supplementary material for: Chromosome 1 trisomy confers resistance to aureobasidin A in Candida albicans
Source: Front Microbiol. 2023 Mar 17;14:1128160. doi: 10.3389/fmicb.2023.1128160 (PMC10063858; doi:10.3389/fmicb.2023.1128160)
Supplement: Supplementary file 2 [file Table_2.DOCX]

**Table S2. Sequences of primers used in this study**

| Primer name | Primer sequence (5' to 3') |
| --- | --- |
| Gene deletions | |
| BP1639 | CAGATCGTACAATAAAGCTTTGAAG |
| BP1640 | TGCGTCTATTTATGTAGGATGAAAG |
| CaPDR16-US-F | GGTAGCAATGGCAGCAG |
| NAT1-CaPDR16-US-R | GTATAGGAACTTCCTCGAGGGGTGGAGAGACAAGGGTTTATTAG |
| NAT1-CaPDR16-DS-F | AGATCCACTAGTTCTAGAGCGGCATTACATATAGAGAAAACCCCC |
| CaPDR16-DS-R | CAGTACAAATTTATGAATTGGGAG |
| CaAUR1-US-F | TGGATTGAGGCACACTACC |
| NAT1-CaAUR1-US-R | GTATAGGAACTTCCTCGAGGGGTATTGGTGGAATTGTGGTTC |
| NAT1-CaAUR1-DS-F | AGATCCACTAGTTCTAGAGCGGGAGTTCATTTGCTGTTCATTG |
| CaAUR1-DS-R | GACTCCAGTTTCCACTACTACAGG |
| Diagnostic PCR for deletions | |
| CaPDR16-USD-F | GTGATGGAAAGACGTGTCG |
| CaPDR16-DSD-R | CGGAAGTAATTCTCAACATAATTTC |
| CaAUR1-USD-F | GGTGGTGTGTGTGTGTGTG |
| CaAUR1-DSD-R | GTAGCATTAACACTGCTTTCTTG |
