# Supplementary material for: Chromosome 1 trisomy confers resistance to aureobasidin A in Candida albicans
Source: Front Microbiol. 2023 Mar 17;14:1128160. doi: 10.3389/fmicb.2023.1128160 (PMC10063858; doi:10.3389/fmicb.2023.1128160)
Supplement: Supplementary file 3 [file Table_3.DOCX]

Table S3. Relative expression of some genes associated with drug tolerance

| Gene | Ratio (Chr1x3/SC5314) |
| --- | --- |
| Genes associated with aureobasidin A tolerance | |
| *PDR16* | 1.67^*^ |
| *AUR1* | 1.54^*^ |
| Genes associated with caspofungin tolerance | |
| *GSC1* | 0.91 |
| *GSL1* | 1.54^*^ |
| *GSL2* | 0.76^*^ |
| *MKK2* | 0.75^**^ |
| *MKC1* | 0.74^**^ |
| *CMP1* | 1.16 |
| *CNB1* | 0.97 |
| *CRZ1* | 0.91 |
| Genes associated with 5-flucytosine tolerance | |
| *FUR1* | 0.88 |
| *FCY21* | 1.05 |
| *FCY2* | 0.84 |
| *FCA1* | 0.74^*^ |

*q<0.05; **q<0.001
